# Molecular Analysis of a Mutated FSH Receptor Detected in a Patient with Spontaneous Ovarian Hyperstimulation Syndrome

**DOI:** 10.1371/journal.pone.0075478

**Published:** 2013-09-13

**Authors:** Sayaka Uchida, Hiroshi Uchida, Tetsuo Maruyama, Takashi Kajitani, Hideyuki Oda, Kaoru Miyazaki, Maki Kagami, Yasunori Yoshimura

**Affiliations:** Department of Obstetrics and Gynecology, Keio University School of Medicine, Tokyo, Japan; University of Cordoba, Spain

## Abstract

Spontaneous ovarian hyperstimulation syndrome (sOHSS) is a rare event that may result from a FSH-producing pituitary adenoma (FSHoma), activating mutations of the FSH receptor (FSHR), and cross-reactivity of the FSHR to elevated hCG and TSH in the setting of pregnancy or hypothyroidism. The objective of this study was to investigate whether an aberrant FSHR was present in a woman with sOHSS and a non-surgically diagnosed FSHoma whose serum FSH levels and FSH bioactivity were nearly normal. Sequencing of the patient’s *FSHR* gene revealed a heterozygous novel missense mutation c. 1536G>A resulting in an amino acid substitution M512I. We asked whether this mutant FSHR affected FSHR-mediated signaling pathways involving cAMP/protein kinase A (PKA), phosphatidylinositol-3 kinase (PI3K)/protein kinase B (AKT) and v-src sarcoma (Schmidt-Ruppin A-2) viral oncogene homolog kinase (SRC)/ p42/p44 extracellular signal-regulated protein kinases (ERK1/2). Thus, 293T cells expressing wild-type (FSHRwt), the mutant FSHR (FSHRmt), or both (FSHRwt/mt) were treated with FSH and subjected to measurements of intracellular cAMP, cAMP-induced CRE (cAMP response element)-mediated luciferase assays and immunoblot analyses of phosphorylated PI3K and ERK1/2. There were no differences in luciferase activities or phosphorylation levels of ERK1/2 among FSHRwt, FSHRmt cells and FSHwt/mt cells. However, FSHRmt cells showed a significant reduction in both cAMP production and PI3K phosphorylation levels with unchanged phosphorylation of ERK1/2 upon FSH stimulation in comparison to FSHwt cells. Also, FSH treatment did not provoke PI3K phosphorylation in FSHwt/mt cells. These results indicate that the novel missense M512I FSHR mutation identified herein did not participate in hyperactivation of FSHR-mediated signaling pathways but rather in hypoactivation of the FSH-mediated PI3K/AKT pathway. Thus, this study demonstrates a new functional property of this novel mutatnt FSHR, which, however, might not be involved in the pathogenesis of sOHSS in this FSHoma patient.

## Introduction

Ovarian hyperstimulation syndrome (OHSS) is most often an iatrogenic complication of ovulation induction and controlled ovarian hyperstimulation using gonadotropin(s) in infertile patients. Administration of excess recombinant/urinary FSH stimulates the growth of multiple follicles, which results in the hyperproduction of estrogens. The subsequent LH/hCG administration for triggering ovulation is the inciting factor for OHSS. OHSS is characterized by marked ovarian enlargement and increased vessel permeability. It results from the production of bioactive substances such as vascular endothelial growth factor. Moderate to severe OHSS occurs in 0.2% to 2% of all ovarian stimulation cycles [1,2]. Severe OHSS is a life-threatening complication accompanied by massive ovarian enlargement, ascites, pleural effusion, oliguria, hemoconcentration, and thromboembolism. Very rarely, patients may manifest signs of OHSS spontaneously during pregnancy or as a result of hypothyroidism, a condition referred to as spontaneous ovarian hyperstimulation syndrome (sOHSS) [3,4]. Cases of sOHSS together with its possible underlying mechanisms have been reported, and include the cross-reactivity of the FSH receptor (FSHR) to a pregnancy- or hypothyroidism-induced elevation of hCG, in response to TSH in which the specific β-subunits are similar to that of FSH [5-7], activating mutations of the *FSHR* [5-7], or hypergonadotropinemia caused by gonadotropin-producing pituitary adenomas [8-26]. Among these pituitary adenomas, reproductive-aged women with an FSH-producing adenoma (FSHoma) exhibit sOHSS characterized by high serum levels of FSH and estradiol (E_2_) together with enlarged multicystic ovaries [8-15]. A plausible explanation is that the excess FSH is the cause of sOHSS; however, we, and others have reported cases of FSHoma-associated sOHSS in which serum FSH levels are nearly normal. In some of these cases E_2_ was elevated; though this was not universal [17-26]. Thus, the mechanism by which an FSHoma induces OHSS is not fully understood. Intriguingly, it has been reported that the FSH produced by an FSHoma possesses enhanced bioactivity, thereby provoking OHSS even when within the normal range [19,27,28]. We previously reported a woman with a non-surgically diagnosed FSHoma and sOHSS, whose serum FSH levels were only mildly elevated [22]. We, then assayed the bioactivity of the serum FSH from this patient and found it to be similar to the activity in control women [29]. In the present study we identified a novel missense mutation of the FSHR in the same patient and performed an *in vitro* functional analysis on the mutated FSHR to elucidate the molecular mechanism(s) underlying sOHSS caused by this FSHoma.

## Materials and Methods

### Ethics Statement

This study was approved by the Institutional Review Board at Keio University School of Medicine (#2011-316). The patient, her mother and two sisters provided written informed consent.

### DNA Sequencing of the FSHR gene

Genomic DNA was extracted from peripheral blood leukocytes of the patient and from the saliva of her mother and two sisters using the DNeasy Blood & Tissue Kit (Qiagen, Tokyo, Japan) and the Oragene DNA Self-Collection kit (DNA Genotek Inc., Ottawa, ON, Canada). All exons of the human *FSHR* gene were sequenced from the patient’s genomic DNA. The sequence of the segment harboring the mutation was determined in the product of two independent polymerase chain reactions (PCRs) using genomic DNA extracted from the patient and her family. We employed two in silico algorithms, PolyPhen [30] and SIFT [31], to predict the putative impact of missense variant(s) on protein function. Scores were classified as tolerated or deleterious according to the proposed criteria [30,31]. We also searched for known SNPs using the NCBI-dbSNP database (build 137) [32] and the Japanese Single Nucleotide Polymorphisms (JSNP) DATABASE (release 40) [33,34].

### Plasmid construction and site-directed mutagenesis

An expression plasmid encoding the human *FSHR* (h*FSHR*), pcDNA3-h*FSHR* (pc*FSHRwt*), was a generous gift from Dr. Aaron J.W. Hsueh, Division of Reproductive Biology, Department of Gynecology and Obstetrics, Stanford University Medical Center. A cAMP-responsive element-driven luciferase reporter plasmid p3 x CRE-Luc was constructed as described previously [29]. Briefly, three copies of oligonucleotides coding for the canonical cAMP responsive element (CRE) derived from the rat somatostatin gene promoter region were subcloned into a firefly luciferase reporter vector pE1b-Luc [29]. A sea pansy luciferase expression vector pRL-SV40 was purchased from Promega (Madison, WI). All plasmids for transfection experiments were prepared using a Genopure Plasmid Maxi Kit (Roche Molecular Biochemicals, Indianapolis, IN). The identified mutation was introduced into the complementary DNA of pc*FSHRwt* using the QuickChange^TM^ Site-Directed Mutagenesis Kit (Agilent Technologies, Inc. Santa Clara, CA), with synthetic oligonucleotide primers containing the desired mutation. The primers used for the mutation were as follows; forward primers 5 ‘C ATC AGC AGC TAC ATA AAG GTG AGC ATC TGC3’ and reverse primer 5’ G TAG TCG TCG ATG TAT TTC CAC TCG TAG ACG3’. The constructed vector harboring the mutated FSHR (pc*FSHRmt*) was verified by sequencing.

### Cell culture, transient transfection, and luciferase assay

The 293T human renal epithelial (293T) cell line was obtained from the American Type Culture Collection (Manassas, VA) and was maintained in Dulbecco’s modified Eagle’s medium, supplemented with 10% fetal bovine serum, 100 U/ml penicillin, 100 µg/ml streptomycin, and 250 ng/mL amphotericin B. Twenty-four hr prior to transfection, 5 x 10^4^ cells were seeded in a 24 well plate. The cells were then transfected with 400 ng of pcDNA3 vector, pc*FSHRwt*, pc*FSHRmt*, or a mixture of equal amounts of pc*FSHRwt* and pc*FSHRmt* together with 400 ng of a cAMP-responsive element-driven luciferase reporter plasmid p3 x CRE-Luc and 4 ng of a sea pansy luciferase expression vector pRL-SV40, using Lipofectamine 2000 Reagent (Life Technologies Co., Carlsbad, CA). Twenty-four hr after transfection, 10 to 100 mIU/mL recombinant human FSH (rhFSH; Gonal-F^®^, Serono, Aubonne, Switzerland), 1 to 50 IU/mL hCG (Sigma-Aldrich, Japan) or serum samples collected from either the patient or a healthy control woman with regular menstrual periods were added. After 24 hr of incubation, the firefly and sea pansy luciferase assays were performed with the dual luciferase assay system (Promega) according to the manufacturer’s protocol. Luciferase activities were determined using a TD-20/20 Luminometer (Turner Designs, Sunnyvale, CA). Firefly luciferase activities were normalized with the sea pansy luciferase activities, and described as relative light units (RLU).

### Immunoprecipitation and immunoblot

293T cells were transfected with either the empty vector, pc*FSHRwt*, pc*FSHRmt*, or an equal mixture of pc*FSHRwt* and pc*FSHRmt*. Twenty four hr after transfection, they were treated with or without 100 mIU/mL FSH for 15 min, washed with PBS on the culture dish, and directly collected and lysed in RIPA buffer (20 mM Tris-HCl pH 7.5, 150 mM NaCl, 1 mM EDTA, 1% Na-deoxycholate, 0.1% SDS, 1 mM Na _3_VO_4_, 50 mM NaF, 1 mM Na _2_MoO_4_). Protein concentrations were determined using a DC protein assay kit (Bio-Rad Laboratories, Hercules, CA) with BSA as a standard. Total cell lysates (250 µg) obtained from 293T cells transfected with vector alone, pc*FSHRwt*, pc*FSHRmt*, or an equal mixture of pc*FSHRwt* and pc*FSHRmt*, were immunoprecipitated using protein G Sepharose 4B (GE Healthcare, Fairfield, CT) human FSHR antibody (Santa Cruz Biotechnology, Inc., Santa Cruz, CA; sc-7798). The immunoprecipitates or 20 µg of the same total lysates were electrophoresed on 8% or 12% SDS-PAGE for 2 hr at 25 mA and subsequently transferred onto a polyvinylidene fluoride membrane (Immobilon-P, Millipore, Bedford, MA) for 2 hr at 50 V. After blocking nonspecific binding in 5% BSA containing TBS-T for 1 hr, the membranes were incubated overnight at 4°C with primary antibodies against the human FSHR, β-actin (Santa Cruz Biotechnology, Inc.), phospho-ERK1/2 (Cell Signaling Technology, Boston, MA), total ERK1/2 (Millipore, Darmstadt, Germany), phospho-PI3K p85 (Tyr458), or total PI3K p85 (Cell Signaling Technology) in TBS-T containing 2% BSA. After washing with TBS-T three times, the membranes were incubated with an appropriate horseradish peroxidase-conjugated secondary antibody (1:10,000) for 1 hr, and washed three times with TBS-T for 10 min. The immunoreactive proteins were detected by the enhanced chemiluminescence method (GE Healthcare). The intensities of protein bands were quantified with ImageJ® software.

### Immunofluorescent study

Prior to transfection, 293T cells were cultured in 8-well chamber glass slides coated with poly L-lysine (Life Technologies Co.). After 24 hr, 293T cells were transfected with either the empty vector, pc*FSHRwt*, pc*FSHRmt*, or an equal mixture of pc*FSHRwt* and pc*FSHRmt* using Lipofectamine 2000. Twenty-four hr after transfection, they were fixed with 3.7% paraformaldehyde. Without permeabilization, cells were incubated with anti-FSHR antibody, followed by staining with Cy3-conjugated anti-goat IgG. Fluorescent images were photographed with a fluorescent microscope, BIOREVO® BZ-9000 (Keyence, Osaka, Japan). The intensity of the FSHR signal at the cell surface of each cell was measured with ImageJ® software. From three independent transfections, FSHR intensities of 30 cells were measured and divided by each cell’s body area.

### Cell proliferation assay

293T cells (1 x 10^4^) were prepared in 96-well culture plates. Twenty-four hr later, the indicated vector was transiently transfected with Lipofectamine 2000 (Day 0). Transfected 293T cell proliferation was measured with the CellTiter96 Aqueous One Solution Cell Proliferation Assay Kit (Promega) according to the manufacturer’s protocol at Day 0, 1, 2 and 3.

### cAMP assay

293T cells were seeded at a density of 1 x 10^4^ cells in 96-well culture plates. The following day, they were transfected with the indicated vector. Two days after transfection, cells were stimulated without or with 10 or 100 mIU/mL FSH for 30 min and were subjected to a cAMP assay using cAMP-Glo Max Assay Kit (Promega). According to the manufacturer’s recommendation, ∆RLU was calculated as the RLU difference between FSH-stimulated and unstimulated cells.

### Statistical analysis

All experimental data from the bioassays represent the results obtained from three independent experiments, expressed as the mean and SEM. Statistical analysis was performed utilizing Student’s *t* test. Immunofluorescent, immunoprecipitation and immunoblotting studies were repeated three times, and representative images are shown. The differences were considered significant at *P* < 0.05.

## Results

### Case report

The clinical aspects of this case have been described previously [22]. Briefly, the patient, a 40-year old nulligravida woman, presented with enlarged multicystic ovaries which had been detected on transabdominal ultrasonography performed during a health screening visit. She had no complaints except for mild menstrual irregularity, with a cycle length ranging from 20 to 50 days.

**Figure 1:**
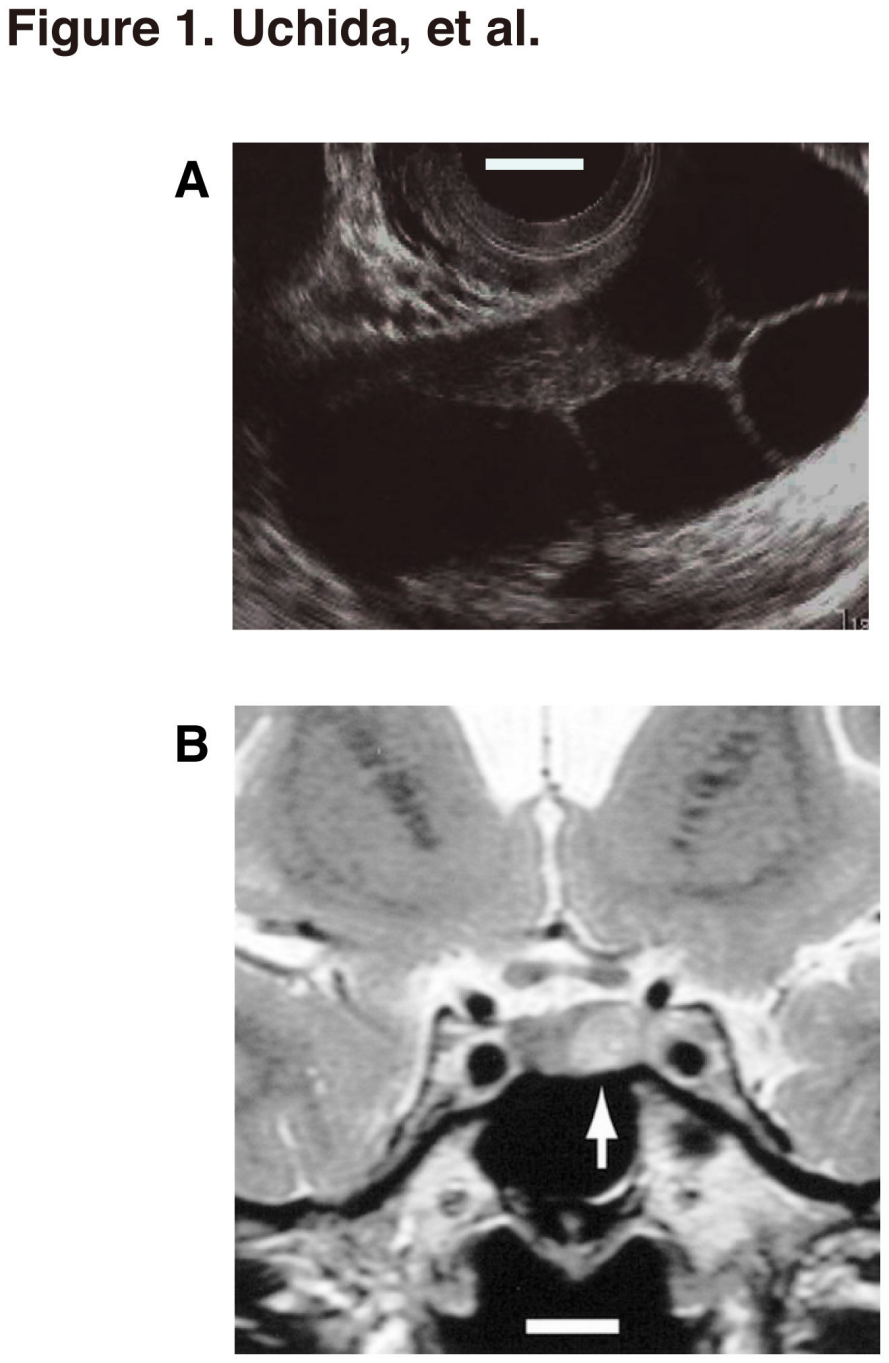
Multiple ovarian cysts and the pituitary microadenoma in the patient. (A) Transvaginal ultrasonographs of the right ovary taken at the initial visit. Bar, 1 cm. (B) T2-weighted coronal magnetic resonance imaging performed 3 months after the initial visit revealing an intra-sellar tumor measuring 9 mm diameter, with a high signal intensity in the left side of the pituitary (arrow). Bar, 1 cm.

On her initial evaluation, the serum E_2_ and prolactin (PRL) concentrations were found to be mildly elevated, ranging as high as 397 pg/mL (normal follicular phase is less than 97) and 24.7 ng/mL (the range for the follicular phase is 2.4 to 18.7), respectively. FSH and LH concentrations were 8.8 IU/L (follicular phase is 2.9 to 12.3) and less than 0.2 IU/L (the range for the follicular phase is 1.4 to 8.6), respectively. The patient was observed for several years without treatment and the ovarian size was monitored by transvaginal ultrasonography (Figure 1A) Over this period, the serum E_2_ concentration fluctuated dramatically (mean ± SD, 503 ± 393 pg/mL; range, 43-1,367 pg/mL) with the menstrual cycle and transiently normalized. Serum FSH levels were normal or mildly elevated (mean ± SD, 11.1 ± 4.8 mIU/mL; range, 6.0-26.1 mIU/mL) throughout the cycle.

T2-weighted coronal magnetic resonance imaging revealed an intra-cellar tumor measuring 9 mm diameter, with a high signal intensity in the left side of the pituitary (arrow, Figure 1B), which remained unchanged 12 months after the initial visit. After the intravenous administration of thyrotropin-releasing hormone (TRH), the patient exhibited paradoxical secretory responses of FSH and LH, which collectively led to a presumed diagnosis of an FSH-producing, pituitary microadenoma (FSHoma) [22].

Since the patient’s serum FSH levels were normal to mildly elevated, we postulated that her sOHSS might result not only from increased FSH levels, but also from increased FSH bioactivity. To address this question, we measured the FSH bioactivity of this patient’s serum using cAMP-responsive element-driven luciferase assays and granulosa cell aromatase assays [29]. FSH bioactivity, however, did not exceed that of control subjects [29]. Thus, the OHSS phenotype in this FSHoma patient was not due to altered FSH bioactivity.

### Identification of a novel M512I missense mutation in the FSHR of the sOHSS patient and her family

The patient presented with sOHSS and a non-surgically diagnosed FSHoma. However, her FSH serum levels were only mildly elevated, and its bioactivity was in the normal range [22,29]. We, therefore, postulated that aberrant FSHR-mediated signaling pathways might be involved in the pathogenesis of sOHSS in this patient. To address this problem, we first examined the DNA sequence of her *FSHR* gene.

**Figure 2:**
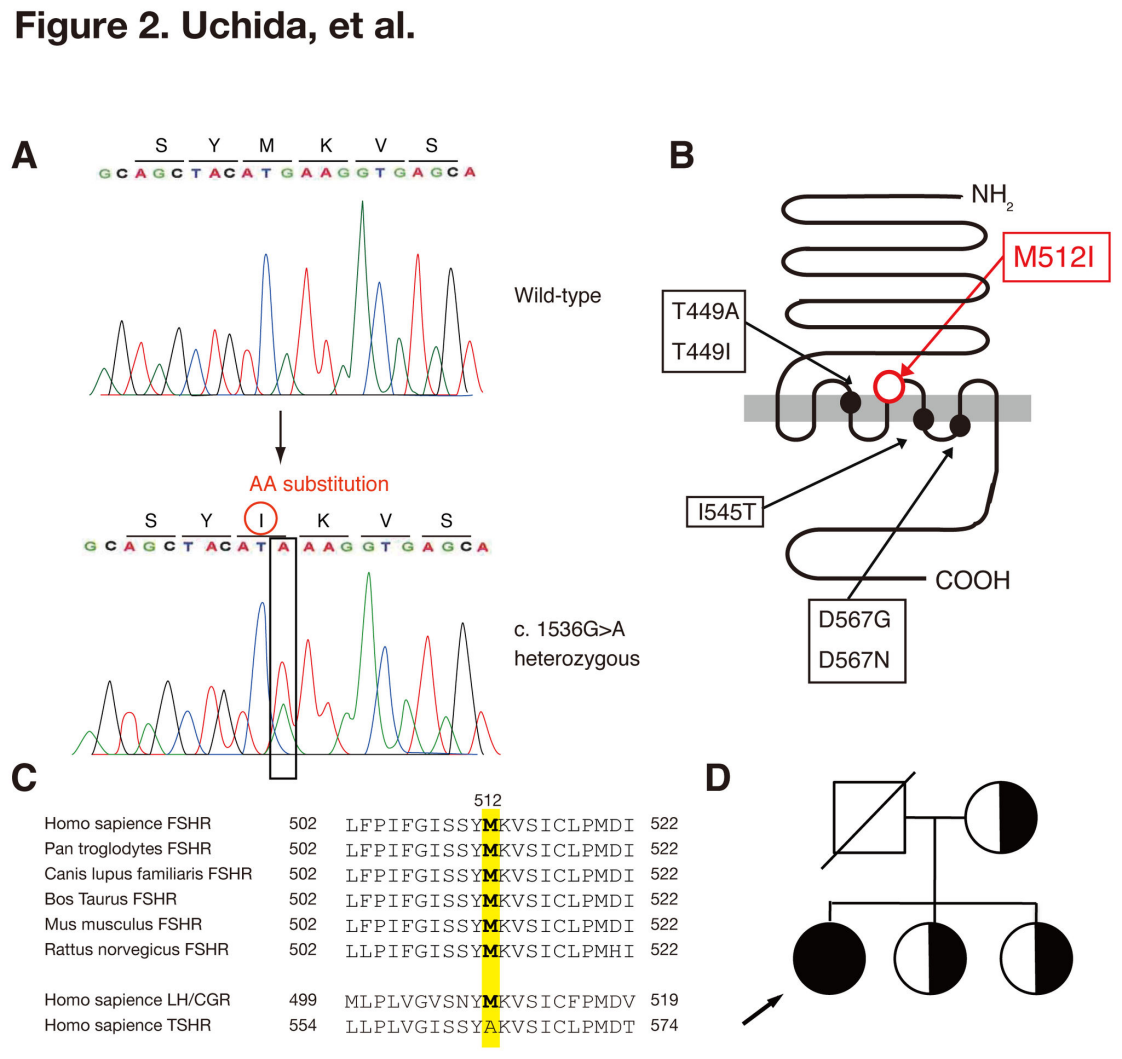
New FSHR mutation and family pedigree. (A) Result of nucleotide-sequencing of exon 10 of the hFSHR of the patient in comparison to the respective wild-type sequence. The transversion G>A at position c.1536 is indicated by an arrow on the wild-type sequence. In-frame amino acids are indicated above each sequence. (B) The location of the amino acid substitution in the FSHR protein. The previously reported activating mutations linked to sOHSS are also indicated [42]. (C) The sequences around the target region of FSHR in other species. The relevant amino acid sequences of human luteinizing hormone receptor (LH) and thyroid-stimulated hormone receptor (TSHR), which are highly homologous to human FSHR as analyzed by Blastp (http://blast.ncbi.nlm.nih.gov/Blast.cg), are also listed. (D) Family pedigree. The arrow indicates the patient. The black symbol indicates the occurrence of the sOHSS symptoms. Half-shaded symbols indicate unaffected heterozygotes. Circles represent females and squares male family members.

Sequencing of all exons of the *FSHR* gene derived from genomic DNA revealed that the patient was heterozygous for a G to A transversion (c.1536G>A) that substituted a methionine (Met, M) for isoleucine (Ile, I) at position 512 (p.Met512Ile, M512I) (Figure 2A), located in the transmembrane region of FSHR (Figure 2B). Met at 512 of FSHR is highly conserved among many species, suggesting essentiality for its structure and function (Figure 2C). No other nucleotide sequence transversion causing an exchange of an amino acid was found in this case. The in silico algorithm SIFT and PolyPhen was consistent with this mutation being deleterious. Additionally, we searched for known SNPs using the NCBI-dbSNP database and the JSNP database [32-34]. No SNPs identical to the 1536G>A mutation were found in either database. This mutation is deposited in DDBJ (www.ddbj.nig.ac.jp) under accession number AB839941. The sequencing of the target region on the *FSHR* gene amplified from the genomic DNA of the patient’s mother and two sisters, revealed that the mutation was maternal in origin and that patient’s mother and two sisters had the same heterozygous mutation (Figure 2D).

### No significant enhancing effect of the mutant FSHR on the FSH/FSHR/cAMP/PKA pathway

**Figure 3:**
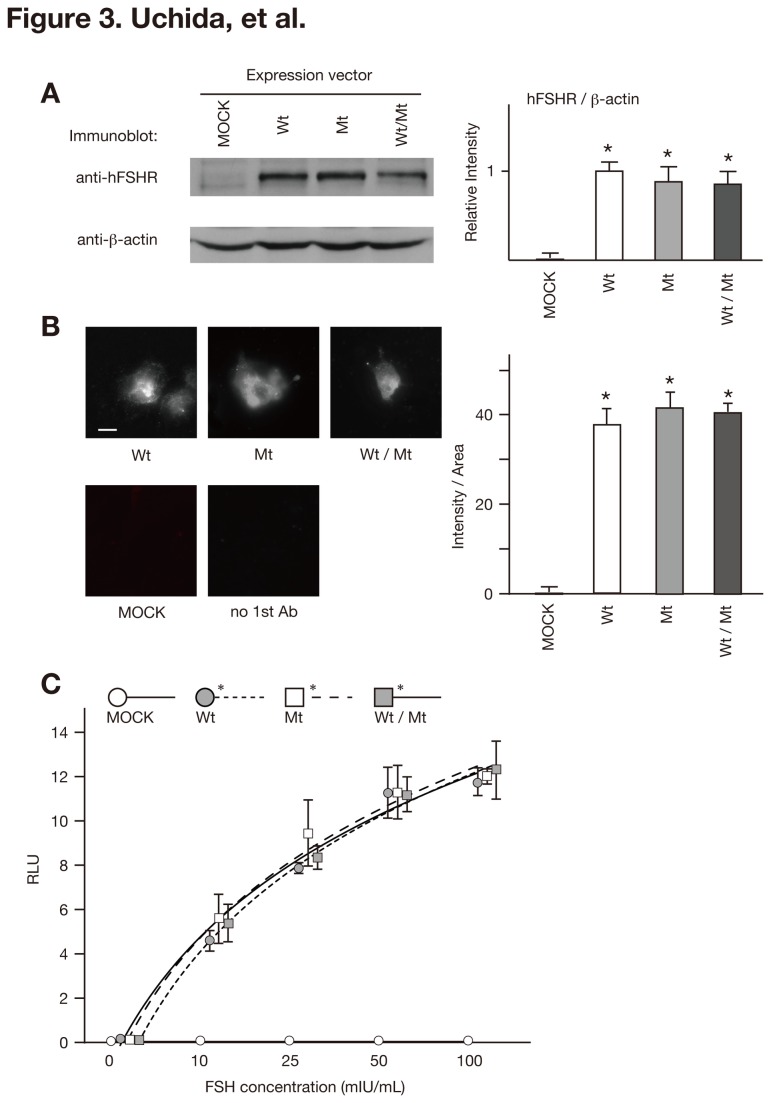
Luciferase activities of FSHRwt, FSHRmt, and FSHRwt/mt cells following treatment with FSH. (A) Immunoblot analysis of FSHR immunoprecipitates (upper panel) and total cell lysates (lower panel) derived from 293T cells transfected with empty vector (MOCK), pc*FSHRwt* (Wt), pc*FSHRmt* (Mt), or an equal mixture of pc*FSHRwt* and pc*FSHRmt* (Wt/Mt) using the indicated antibodies. The band intensity of FSHR was quantified using ImageJ® software (http://rsbweb.nih.gov/ij/). Data are expressed as the mean ± SEM, and the relative intensity of FSHRwt cells was set at 1.0. There were no significant differences in the band intensity among FSHRwt, FSRmt, and FSHwt/mt cells. (B) Immunofluorescent signal of FSHR expressed at the cell surface was obtained from 293T cells transfected with the indicated vector and stained with anti-FSHR antibody without permeabilization. Bar, 20µm. Intensity of fluorescent signal was measured with ImageJ® software and divided by cell body area. Data expressed the mean ± SEM. There were no significant differences in the band intensity among FSHRwt, FSRmt, and FSHwt/mt cells. (C) Luciferase assay. MOCK, FSHRwt, FSHRmt, and FSHRwt/mt cells were treated with 0, 10, 25, 50, or 100 mIU/mL recombinant human FSH and subjected to a luciferase assay. Firefly luciferase activities were normalized with the sea pansy luciferase activities, and described as relative light units (RLU). Each bar indicates the mean ± SEM, obtained from three different independent experiments. Asterisks show the significant difference of each fitted curve compared to MOCK cells (*P*<0.05).

The combination of a missense mutation in the conserved region of the FSHR in this patient with sOHSS, the FSHoma and the result of the in silico analysis led us to postulate that M512I might enable FSHR to hyper-respond to normal or mildly elevated intrinsic FSH. sOHSS then results from hyper-activation of the FSH/FSHR signaling pathway. To address our hypothesis, we prepared wild-type and mutant FSHR expression vectors that were used to transfected 293T cells together with a cAMP-responsive element-driven luciferase reporter plasmid p3 x CRE-Luc. The cells were then subjected to luciferase assays. We first confirmed using an immunoblot analysis and immunofluorescence that there were no differences in the expression levels of the FSHR among 293T cells transfected with pc*FSHRwt* (FSHRwt cells), pc*FSHRmt* (FSHRmt cells), or an equal mixture of pc*FSHRwt* and pc*FSHRmt* (FSHRwt/mt cells) (Figure 3A and 3B, respectively). FSHRwt/mt cells were used as a heterozygote model to reproduce the phenotype of the patient. Luciferase assays revealed that the increase in luciferase activity was dose-dependent and ranged from 1 × 10 to 10^3^ mIU/L FSH in FSHRwt cells (Figure 3C and data not shown), indicating that our assay system could specifically and quantitatively assess the activation of the FSH/FSHR/cAMP-mediated signaling pathway. However, FSHRmt and FSHRwt/mt cells also exhibited a similar FSH-dose response increase, and there were no differences in the luciferase activities among FSHRwt, FSHRmt, and FSHRwt/mt cells (Figure 3C).

### Cross-reactivity of the mutant FSHR to hCG

**Figure 4:**
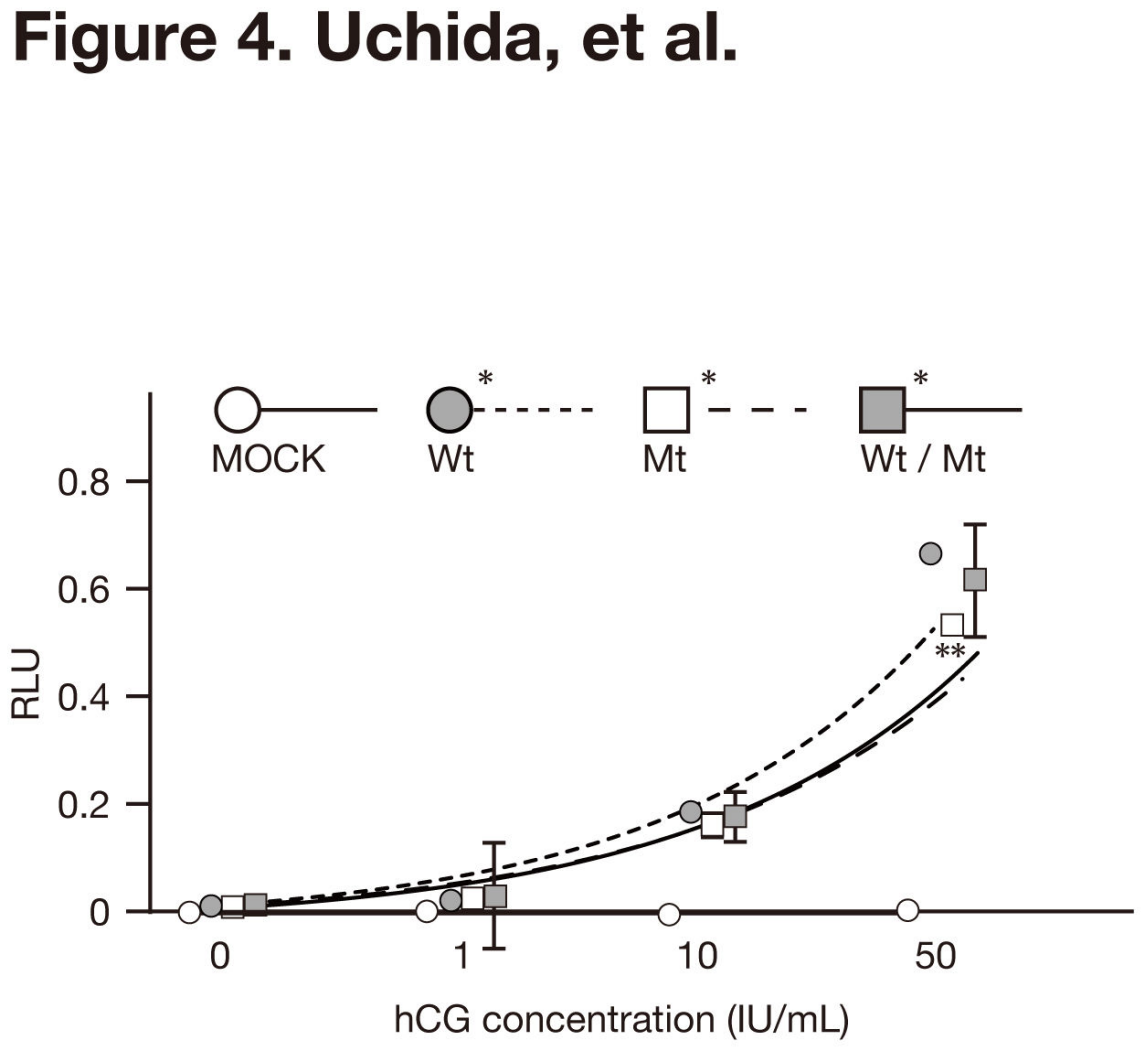
Luciferase activities of FSHRwt, FSHRmt, and FSHRwt/mt cells following treatment with hCG. MOCK, FSHRwt, FSHRmt, and FSHRwt/mt cells were treated with 0, 1, 10, 50 IU/mL hCG and subjected to a luciferase assay. Firefly luciferase activities were normalized with the sea pansy luciferase activities, and described as relative light units (RLU). Each bar indicates the mean ± SEM, obtained from three different independent experiments. Asterisks show the significant difference of each fitted curve compared to MOCK cells. A double-asterisk shows the significant difference compared to FSHRwt cells treated with 50 IU/mL hCG (*P*<0.05).

The gain of cross-reactivity of several types of mutant *FSHR* to hCG and TSH has been suggested as possible causes for sOHSS [5-7]. FSHRwt, FSHRmt and FSHRwt/mt cells exhibited subtle but significant increases in luciferase activities in response to high concentrations of hCG that ranged from 10 to 50 IU/mL (Figure 4). Compared to FSHRwt cells, FSHRmt cells showed a marginally but significantly lower response to high concentrations of hCG (50 IU/mL).

### The patient’s serum interacted normally with the wild-type and rather negatively with mutant FSHR

**Figure 5:**
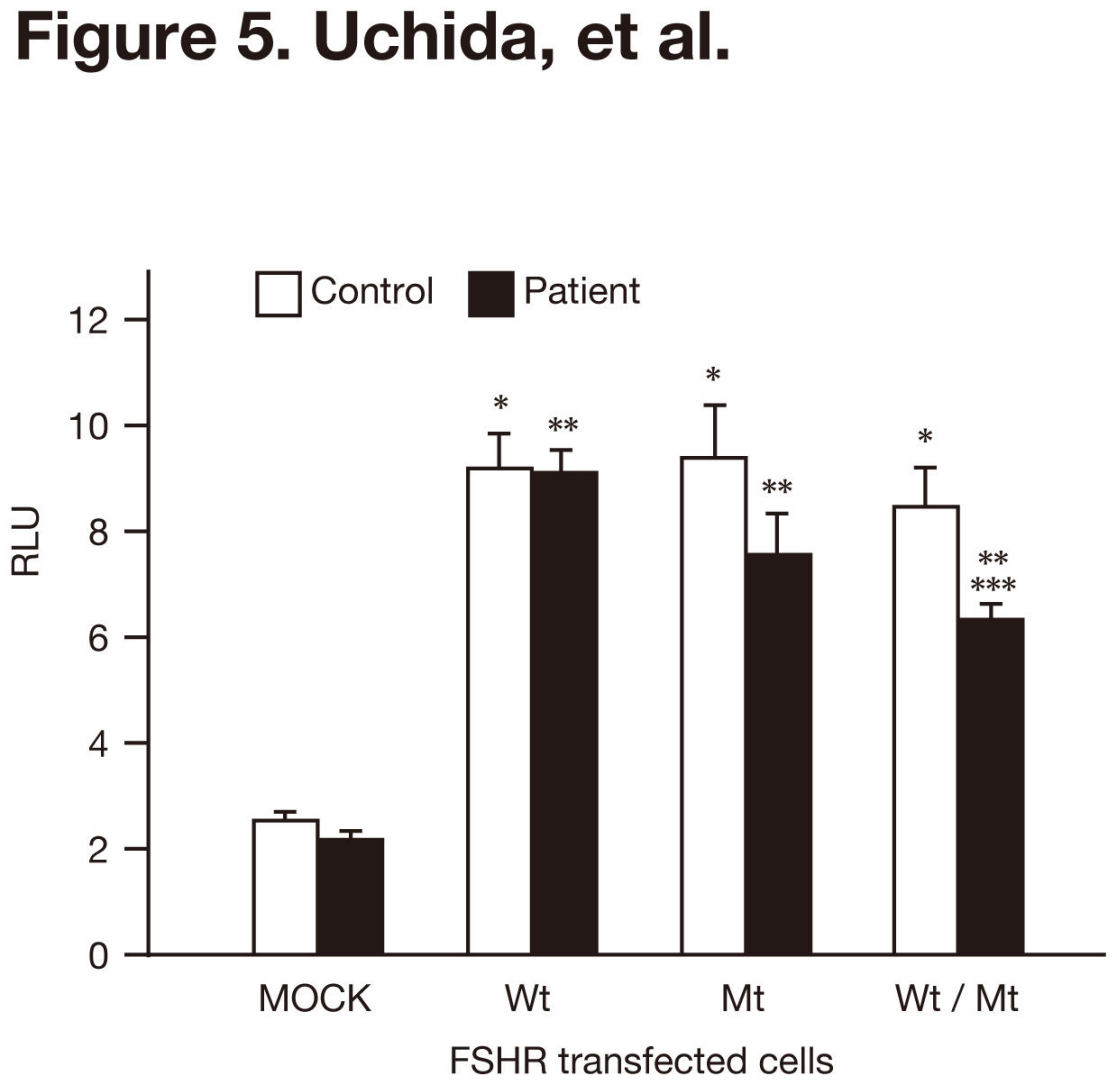
Luciferase activities of FSHRwt, FSHRmt, and FSHRwt/mt cells following treatment with FSH in combination with the patient’s serum. MOCK, FSHRwt, FSHRmt, and FSHRwt/mt cells were treated without and with 100 mIU/mL FSH in combination with control or the patient’s serum and subjected to a luciferase assay. Firefly luciferase activities were normalized with the sea pansy luciferase activities, and described as relative light units (RLU). Each bar indicates the mean ± SEM, obtained from three different independent experiments. Asterisks, double asterisks and a triple asterisks show the significant difference compared to MOCK cells treated with control serum, MOCK cells treated with patient’s serum, and FSHRwt treated with patient’s serum, respectively (*P* <0.05).

We have previously reported that the serum FSH bioactivity of this patient is similar to that of a normal control patient [29]. In the previous experiments we used CHO-K1 cells and rat granulosa cells, both of which expressed the wild-type FSHR, to test the FSH bioactivity of the patient’s sera [29]. A possibility is that the patient’s serum may stimulate the mutant FSHR differently from the wild-type FSHR. To address this possibility, we performed luciferase assays in the presence of the serum derived from the patient or a healthy control woman who had regular menstrual cycles. In contrast to our expectation, both produced similar luciferase activities in FSHRwt and FSHRmt cells (Figure 5). FSHRwt/mt cells displayed a marginally but significantly lower luciferase activity in the presence of the patient’s serum than in the control serum (Figure 5). These data were the opposite of our expectations. The results suggested that there might not be positive interactions between the mutated FSHR and the patient’s serum.

### Impairment of cAMP production in mutant FSHR-expressing cells

**Figure 6:**
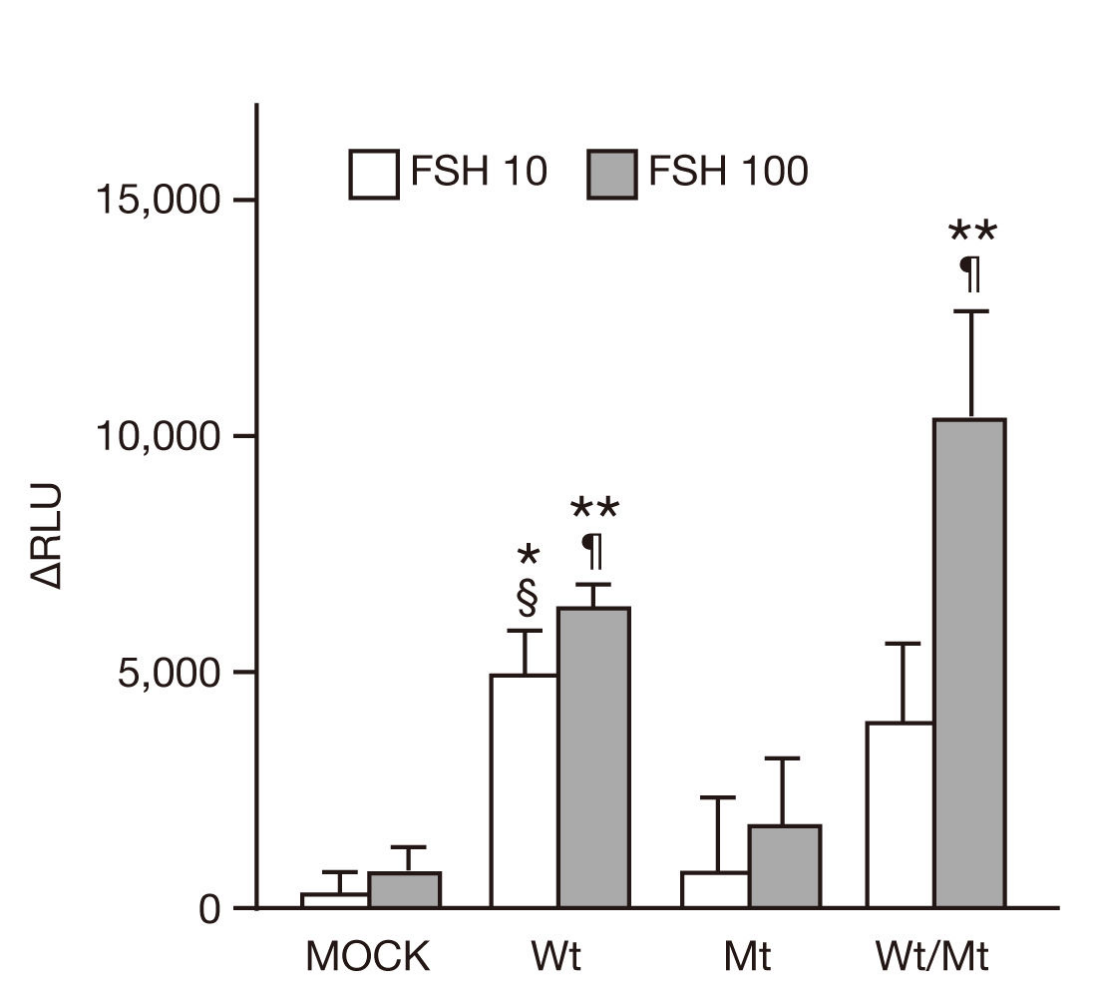
Intracellular cAMP levels in FSHRwt, FSHRmt, and FSHRwt/mt cells following treatment with FSH. Intracellular cAMP levels in 293T cells transfected with empty vector, FSHRwt, FSHRmt, or FSHRwt/mt followed by treatment without and with FSH at 10 mIU/mL (FSH 10) or 100 mIU/mL (FSH 100). Results were obtained with the cAMP-Glo Max assay kit and are shown as ∆relative light unit (∆RLU), the difference between treatment with and without FSH. Data expressed the mean ± SEM, obtained from three independent experiments. Single and double asterisks show the significant difference compared to MOCK cells treated with 10 mIU/mL and 100 mIU/mL of FSH, respectively (*P* <0.05). Two symbols, § and ¶, show the significant difference compared to FSHRmt cells treated with 10 mIU/mL and 100 mIU/mL of FSH, respectively (*P* <0.05).

We found no apparent significant differences in the FSH-induced, CRE-mediated luciferase activities reflecting the FSH/FSHR/cAMP/PKA signaling pathways (Figure 3C). However, FSH/FSHR/cAMP-mediated pathways are known to have at least two branches: the canonical cAMP/PKA/CREB (CRE-binding protein) branch and the cAMP/PKA/GAB2 (adaptor growth factor receptor bound protein 2-associated binding protein 2)/PI3K/AKT branch [35,36]. Therefore, we investigated whether the mutant FSHR affected intracellular cAMP production, an upstream event in the FSH/FSHR/cAMP/PKA pathway. Measurement of intracellular cAMP levels revealed that the level of cAMP was significantly lower in FSHmt cells than in FSHwt cells following FSH-stimulation (Figure 6). In contrast, there were no significant differences in intracellular cAMP levels between FSHwt and FSHwt/mt cells.

### Impairment of the PI3K/AKT pathway, but not the SRC/ERK1/2 pathway in mutant FSHR-expressing cells

**Figure 7:**
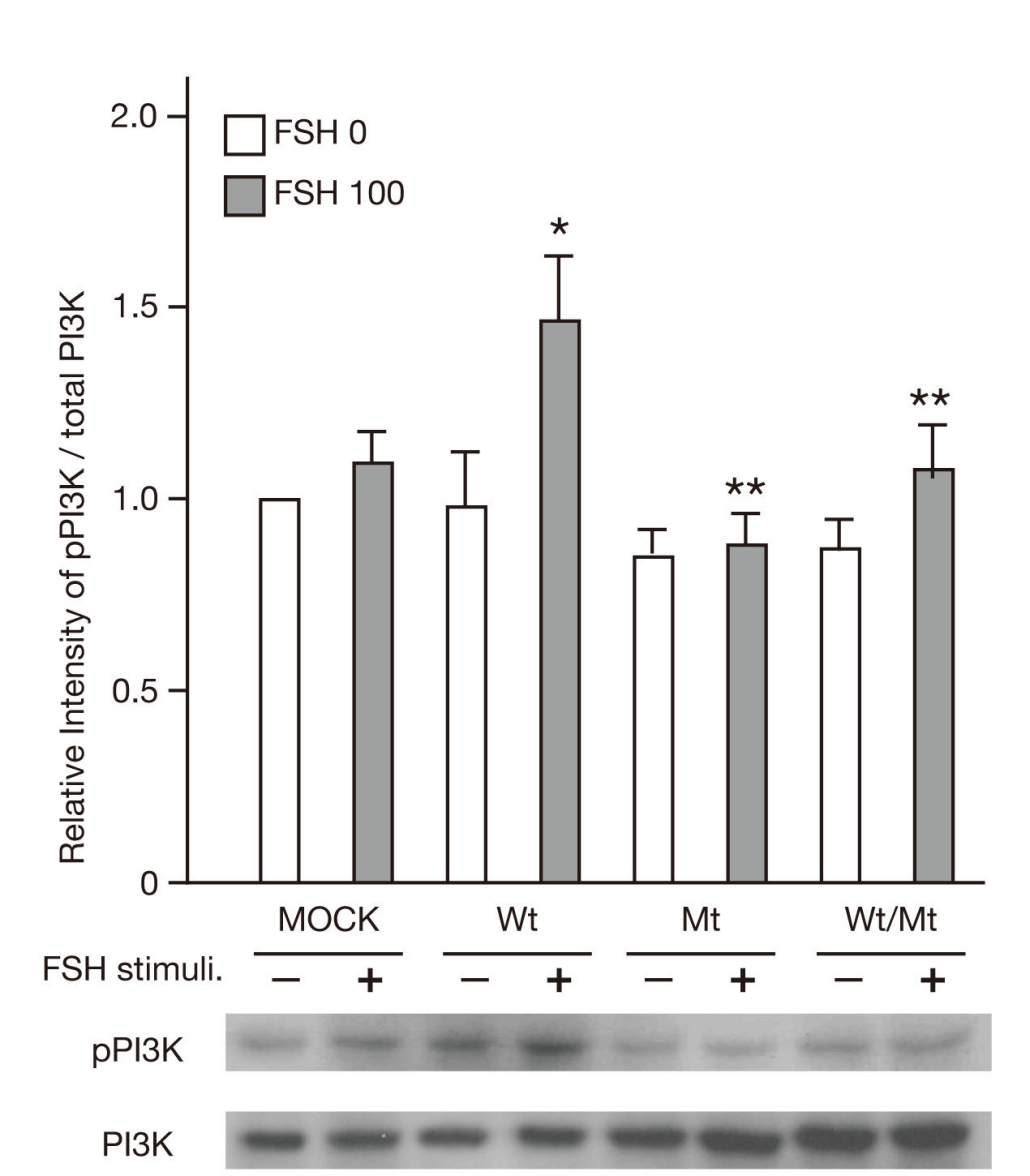
Phosphorylation of PI3K in FSHRwt, FSHRmt, and FSHRwt/mt cells following treatment with FSH. Phosphorylation of PI3K p85 in 293T cells transfected with empty vector, FSHRwt, FSHRmt, or FSHRwt/mt followed by treatment without (FSH 0) and with 100 mIU/mL FSH (FSH 100) were analyzed by immunoblotting using antibodies against phospho-PI3K p85 and total PI3K p85. Each band was quantified with ImageJ® software. Phosphorylation level of PI3K p85 was normalized to the intensity of the total PI3K p85 expression band. Data express the mean ± SEM, obtained from three independent experiments. Single and double asterisks show the significant difference compared to FSHRwt cells treated without and with FSH, respectively (*P* <0.05).

**Figure 8:**
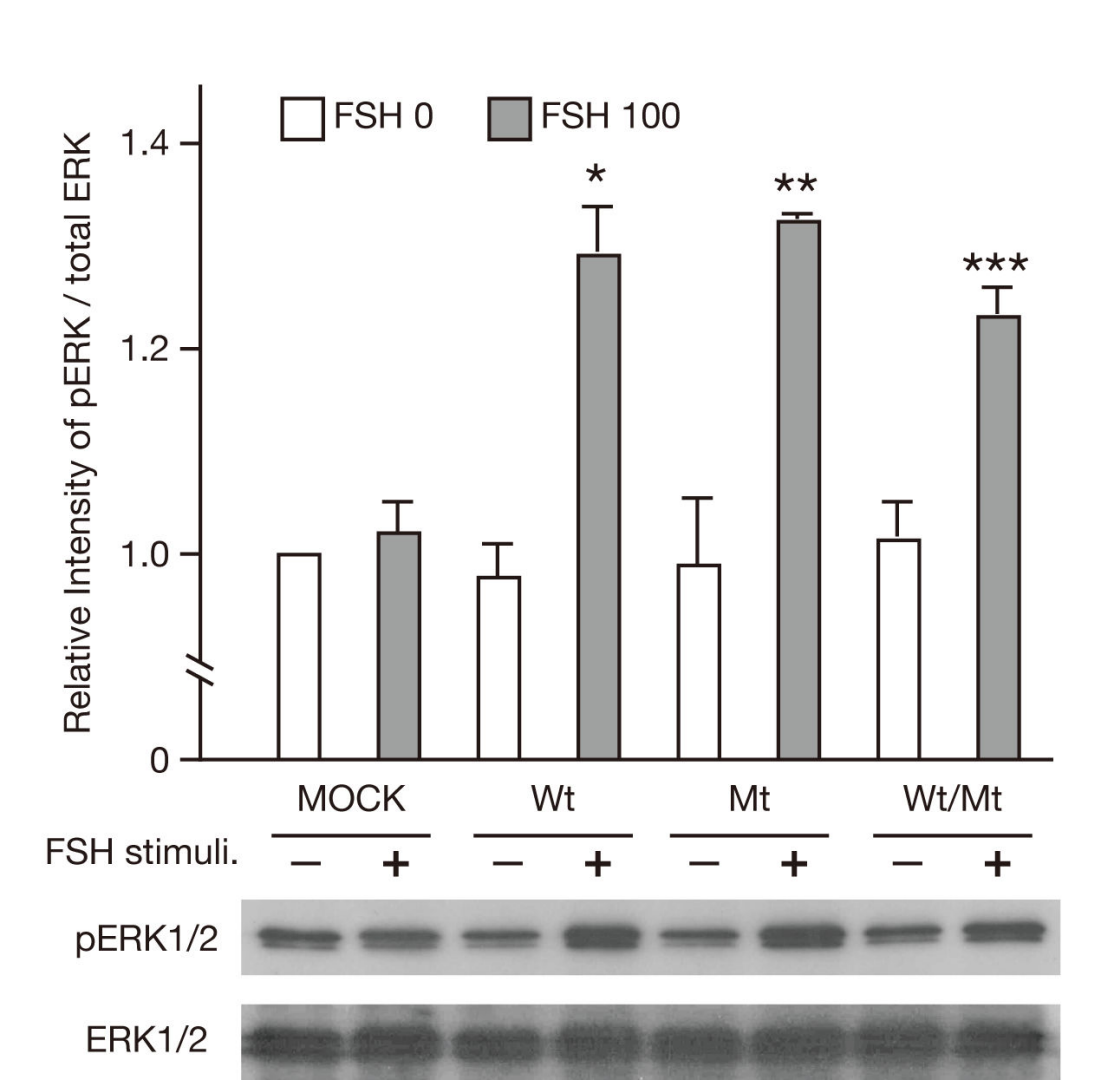
Phosphorylation of ERK1/2 (p42/44 ERK) in FSHRwt, FSHRmt, and FSHRwt/mt cells following treatment with FSH. Phosphorylation of ERK1/2 in 293T cells transfected with empty vector, FSHRwt, FSHRmt, or FSHRwt/mt followed by treatment without (FSH 0) or with 100 mIU/mL FSH (FSH 100) were analyzed by immunoblotting using antibodies against phospho-ERK1/2 and total ERK1/2. Each band was quantified with ImageJ® software. Phosphorylation level of ERK1/2 was normalized to the the intensity of total ERK1/2 expression band. Data express the mean ± SEM, obtained from three independent experiments. Single, double and triple asterisks show the significant difference compared to non-stimulated FSHRwt, FSHRmt and FSHRwt/mt cells, respectively (*P*<0.05).

PI3K/AKT is one of the FSH/FSHR-mediated signaling pathways. We therefore examined whether mutant FSHR influenced the phosphorylation of PI3K and ERK1/2 upon FSH stimulation. As shown in Figure 7, treatment with 100 mIU/L FSH induced phosphorylation of PI3K in FSHwt cells but not in either FSHmt or FSHwt/mt cells. Those results suggested that the FSHR mutant might inhibit FSH-induced activation of the PI3K/AKT signaling pathway. In contrast, ERK1/2 was phosphorylated similarly in FSHwt, FSHmt and FSHwt/mt cells when treated with 100 mIU/L FSH (Figure 8).

### Growth impairment of mutant FSHR-expressing cells by FSH

**Figure 9:**
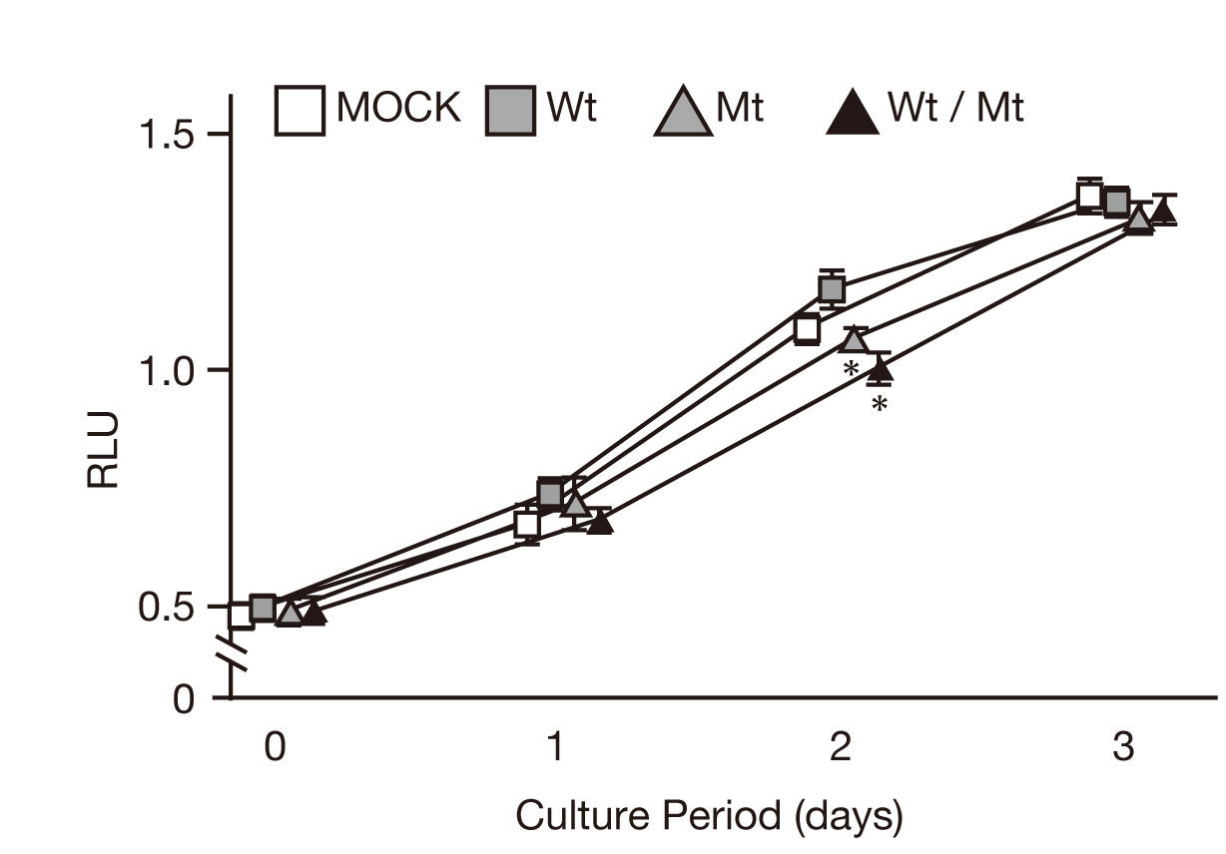
Proliferation of FSHRwt, FSHRmt, and FSHRwt/mt cells following treatment with FSH. Proliferation activities of 293T cells transfected with the indicated vector followed by treatment with 100 mIU/mL FSH for the indicated days. Growth was measured using the CellTiter96 Aqueous One Solution Cell Proliferation Assay Kit and described as relative light unit (RLU). Data show the mean ± SEM, obtained from three independent experiments. Asterisks show the significant difference compared to FSHRwt cells after two days of culture (*P*<0.05).

FSH drives cell proliferation, differentiation and steroid production in granulosa cells [7]. Therefore, we investigated how the expression of the wild-type and mutant FSHR affected FSH-mediated cell proliferation. As shown in Figure 9, inhibition of cell proliferation was observed after 2 days of cultivation of FSHmt and FSHwt/mt cells compared to FSHwt cells.

## Discussion

FSHR belongs to the glycoprotein hormone receptor family, which includes the thyrotropin (TSHR) and lutropin/chorionic gonadotropin (LH/CGR) receptors. These receptors are composed of three distinct domains: 1) a large N-terminal extracellular domain (ECD), 2) a serpentine region containing seven transmembrane segments connected by three extracellular loops and three intracellular loops, and 3) a C-terminal tail that is predicted to be located intracellularly [5,7,37]. The presence of seven transmembrane segments indicates that the gonadotropin receptors are members of the superfamily of G protein-coupled receptors (GPCR). The GPCR superfamily is divided into several major subfamilies, and the glycoprotein hormone receptors belong to the rhodopsin receptor–like subfamily of GPCRs [5,7,37].

The FSHR is expressed in the granulosa cells of the ovary and its activation upon binding of ligands, usually FSH, promotes folliculogenesis. One of the primary and well-characterized signaling cascades results in the activation of adenyl cyclase and the production of the second messenger, cAMP. cAMP, in turn, activates cAMP-dependent protein kinase [protein kinase A (PKA)], leading to the phosphorylation of key substrates, one of which is CREB (cAMP-regulatory element binding protein) [35,37].

Since an *FSHR* mutation was first identified in a patient with sOHSS in 2003 [38], several types of *FSHR* mutations associated with sOHSS have been reported (Figure 2B) [5,7,39]. Most of the mutations are located in the serpentine domain of the FHSR [5], suggesting the gain of cross-reactivity to other ligands of the glycoprotein hormone family, such as hCG or TSH. These hormones consist of a common α-subunit and specific β-subunits that share more than 40% amino acid sequence homology [5]. The cross-reaction is thought to result from the loss of specificity that lowers the intramolecular barrier to activation rather than an increase in binding affinity [5,6]. As such, the FSHRwt would be unable to respond to the low affinity interaction of hCG or TSH with its ECD. In OHSS mutants, the intramolecular barrier to activation would be lower, thus allowing even poor agonists (such as hCG or TSH) to be effective. The novel M512I FSHR mutation found in the present case was also located in the serpentine domain, suggesting that this mutant FSHR may cross-react with hCG. Contrary to our expectation, however, the *in vitro* functional analysis revealed that the mutant FSHR exhibited no specific responses to hCG at 1 IU/mL. The higher doses of hCG (10 and 50 IU/mL) provoked subtle but significant elevation of CRE-mediated luciferase activities in FSHRwt, FSHRmt and FSHRwt/mt cells. However, FSHRmt cells exhibited less luciferase activation compared to FSHRwt cells following treatment with 50 IU/mL FSH. Thus, mutant FSHR did not behave as an activating mutant. Instead, it might be able to act as an inactivating mutation in the presence of high doses of hCG.

In addition to this patient, her mother and two sisters had the same heterozygous mutation but had no symptoms of sOHSS. These findings make it unlikely that this mutation is causative for sOHSS. FSHoma is an extremely rare disease [40], and it is highly unlikely that her mother and two sisters had FSHoma. Given that the mutant FSHR might be preferentially activated in response to only modestly increased FSH levels and/or unknown follicle growth-stimulating factors produced by the FSHoma, it is reasonable that only patients with FSHoma may experience sOHSS. To address this possibility, we examined the activity of this mutated FSHR *in vitro*, in particular, in the presence of the patient’s serum. The wild-type and mutant FSHR, however, exhibited very similar cAMP/PKA activation in response to the FSH contained in the patient’s serum.

The series of negative results prompted us to perform molecular analysis of the FSH/FSHR-mediated signaling pathways other than FSH/FSHR/cAMP/PKA/CREB pathway. First, we focused on intracellular cAMP production, one of the most upstream events of FSH/FSHR signaling pathways. We found that cAMP levels were not increased but were in fact decreased in FSHRmt cells when treated with FSH (Figure 6). This result was contrary to our initial expectation that mutant FSHR might have increased cAMP production and thereby activated its downstream pathway.

We then investigated the FSH/FSHR/PI3K/AKT pathway and found that mutant FSHR inhibited the phosphorylation of PI3K. It has been reported that cAMP activates PKA, which, in turn, induces the phosphorylation of PI3K through GAP2 inactivation in granulosa cells [36]. Given that the mutant FSHR did not produce cAMP upon FSH stimulation (as presented herein), it is possible that mutant FSHR did not increase intracellular levels of cAMP and therefore neither activated PKA nor PI3K. Alternatively, there might be a cAMP-independent mechanism because FSHRwt/mt cells exhibited cAMP production but did not display PI3K phosphorylation upon FSH stimulation. Those findings suggest that mutant FSHR might inhibit PI3K activation directly or at least without involvement of cAMP. PI3K is involved in the proliferation, differentiation, survival and enhanced mRNA translation in granulosa cells [36]. Thus, inhibition of FSH-induced PI3K activation in FSHRmt and FSHRwt/mt cells might contribute to decreased cell proliferation at a specific time point (Figure 9). Elucidation of the precise mechanism awaits further studies.

The other conflicting result is that CRE-mediated luciferase activity was increased without an elevation of cAMP production upon FSH treatment of FSHwt/mt cells. Genua et al. reported an alternative pathway in which ERK1/2 phosphorylated CREB in a cAMP-independent manner and thereby activated CRE-mediated gene transcription [41]. Given the comparable phosphorylation of ERK1/2 in FSHmt cells upon FSH treatment, it is conceivable that the same cAMP-independent ERK1/2-mediated pathway could be activated in FSHwt/mt cells.

In summary, we obtained novel findings regarding the missense M512I FSHR mutation that is newly identified here. This mutant appears to be inactive in that it inhibited FSH-induced cAMP production and PI3K activation. This finding, however, does not support but rather contradicts our initial hypothesis that the mutant FSHR might be an active mutant and, therefore, might hyper-stimulate ovaries in response to the nearly normal or slightly elevated levels of FSH produced from FSHoma in this patient. Although the mechanism underlying OHSS observed in this patient remains to be clarified, we have for the first time demonstrated the presence of a novel mutation of FSHR together with its new properties as an inactive mutant.
